# Effect of cerebral small vessel disease on the integrity of cholinergic system in mild cognitive impairment patients: a longitudinal study

**DOI:** 10.1007/s00415-024-12218-2

**Published:** 2024-02-21

**Authors:** Tiantian Qiu, Hui Hong, Qingze Zeng, Xiaopei Xu, Yanyan Wang, Lixin Zhu, Lige Zhang, Kaicheng Li, Shouping Dai, Xiaodong Li, Fei Xie, Yusong Zhang, Xiao Luo, for the Alzheimer’s Disease Neuroimaging Initiative

**Affiliations:** 1 Department of Radiology, Linyi People’s Hospital, Linyi, China; 2 Department of Radiology, The Second Affiliated Hospital of Zhejiang University School of Medicine, Hangzhou, China; 3 Laboratory Medicine Center, Linyi People’s Hospital, Linyi, China; 4 Department of Equipment and Medical Engineering, Linyi People’s Hospital, Linyi, China

**Keywords:** Cerebral small vessel disease, Cholinergic system, Nucleus basalis of Meynert, Alzheimer’s disease, Magnetic resonance imaging

## Abstract

We aimed to investigate the effect of cerebral small vessel disease (SVD) on cholinergic system integrity in mild cognitive impairment (MCI) patients. Nucleus basalis of Meynert (NBM) volume and cholinergic pathways integrity was evaluated at baseline, 1-, 2-, and 4-year follow-ups in 40 cognitively unimpaired (CU) participants, 29 MCI patients without SVD, and 23 MCI patients with SVD. We compared cholinergic markers among three groups and examined their associations with SVD burden in MCI patients. We used linear mixed models to assess longitudinal changes in cholinergic markers over time among groups. Mediation analysis was employed to investigate the mediating role of cholinergic system degeneration between SVD and cognitive impairment. Increased mean diffusivity (MD) in medial and lateral pathways was observed in MCI patients with SVD compared to those without SVD and CU participants. Both MCI groups showed decreased NBM volume compared to CU participants, while there was no significant difference between the two MCI groups. Longitudinally, compared to CU participants, MCI patients with SVD displayed a more rapid change in MD in both pathways, but not in NBM volume. Furthermore, SVD burden was associated with cholinergic pathway disruption and its faster rate of change in MCI patients. However, mediation analyses showed that cholinergic pathways did not mediate significant indirect effects of SVD burden on cognitive impairment. Our findings suggest that SVD could accelerate the degeneration of cholinergic pathways in MCI patients. However, they do not provide evidence to support that SVD could contribute to cognitive impairment through cholinergic system injury.

**Supplementary Information:**

The online version contains supplementary material available at 10.1007/s00415-024-12218-2.

## Introduction

Alzheimer's disease (AD) is the most common cause of dementia in the elderly and involves multiple pathological processes. In addition to well-established amyloid accumulation, cerebral small vessel disease (SVD) has also been a crucial factor influencing the development of AD [1]. Notably, the presence of both amyloid and SVD pathologies has been suggested to be associated with more severe cognitive dysfunction and a faster rate of cognitive decline in patients with mild cognitive impairment (MCI) and AD [2–4], as well as early conversion from MCI to AD [5, 6]. However, the mechanism underlying the effect of SVD on AD progression remains incompletely understood.

Cholinergic deficits play a critical role in the pathogenesis of AD [7, 8]. Early post mortem studies have illustrated that patients with AD experience a significant loss of basal forebrain (BF) cholinergic neurons, particularly in the nucleus basalis of Meynert (NBM) [9, 10]. In vivo imaging studies further support the findings that patients with MCI exhibit a reduction in NBM volume compared to healthy controls, with even more pronounced reductions in AD patients [11–13]. Cholinergic deficits in AD not only involved in cholinergic NBM neurons but also fibers projecting from the NBM to cortical areas. An autopsy study utilizing cholinergic markers identified two major cholinergic pathways, namely the medial and lateral pathways [14]. Further, recent studies have effectively tracked the two cholinergic white matter (WM) pathways in vivo using neuroimaging analysis techniques [15] and have found that the integrity of these pathways is compromised in individuals with subjective cognitive decline, MCI and AD, which is associated with cognitive decline [16–18].

The relationship between SVD and cholinergic deficits has been previously investigated. In subjects without dementia, increased severity of SVD burden is associated with lower cortical acetylcholinesterase (AChE) activity [19]. Patients with vascular dementia have shown decreased acetylcholine levels and reduced AChE activity in cerebrospinal fluid (CSF) [20, 21], and cholinergic therapies could improve cognition functions [22]. Furthermore, losses of cholinergic pathways assessed using the cholinergic pathways hyperintensities scale (CHIPS) were associated with vascular cognitive dysfunction [23, 24]. However, although several studies have demonstrated the associations between SVD and some cholinergic markers (e.g., cerebral AChE activity [25] and substantia innominate/BF volume [26, 27]) in AD, these findings are inconsistent and inadequate. Whether and how SVD impacts the integrity of the cholinergic NBM and WM pathways in AD progression remains to be clarified.

This study aimed to investigate (1) the changes in cholinergic system integrity in MCI patients with and without SVD, and (2) the potential mediating role of cholinergic deficits in the connection between SVD and cognitive impairment. We will achieve these objectives through comprehensive analyses that combines both cross-sectional and longitudinal approaches. Specifically, we focused on two well-established cholinergic markers, namely the NBM volume and mean diffusivity (MD) of WM pathways, which have previously been shown to be sensitive to cholinergic system damage in AD [17]. We hypothesized that MCI patients with SVD would exhibit more severe cholinergic damage than those without SVD. Moreover, SVD may lead to cognitive impairment by accelerating cholinergic deficits.

## Methods

### Study participants

All data used in the current study were from the Alzheimer’s Disease Neuroimaging Initiative (ADNI) database (http://adni.loni.usc.edu/). This ongoing project was launched in 2003 to develop clinical, neuropsychological, and neuroimaging biomarkers for early disease detection and progression monitoring of AD.

ADNI criteria for MCI patients were: (1) subjective memory complaints, either self-reported, reported by a study partner, or reported by a clinician; (2) objective memory loss defined as scoring below an education adjusted cutoff score on delayed recall of the Wechsler Memory Scale-Logical Memory; (3) a mini-mental state examination (MMSE) score equals to or higher than 24 out of 30; (4) a global clinical dementia rating (CDR) score of 0.5; and (5) general cognitive and functional performance sufficiently preserved so that a diagnosis of dementia could not be made by the site physician at the time of screening. ADNI criteria for cognitively unimpaired (CU) participants were: (1) no report of any cognition complaints; (2) a MMSE score equals to or higher than 24 out of 30; and (3) a CDR score of 0.

### Group stratification based on baseline Aβ levels and SVD severity

The amyloid positron emission tomography (PET) images underwent a standardized preprocessing procedure by the ADNI-PET Core. The standardized uptake value ratio (SUVR) was calculated as the average of the uptake values of the frontal, angular/posterior cingulate, lateral parietal, and temporal cortices divided by the mean uptake values in the cerebellum. As previously described [28], baseline Aβ positivity (A +) was defined by a SUVR ≥ 1.11. Following the research framework proposed by Jack et al. [29], MCI patients with A + were included in our study.

The burden of white matter hyperintensities (WMH) was used to reflect SVD severity and was evaluated on baseline T2 fluid‐attenuated inversion recovery (FLAIR) images according to the Fazekas et al. criteria [30]. Participants with moderate/severe WMH burden were labeled as V + (indicating vascular brain injury), while those with mild WMH burden were labeled as V − . Consequently, MCI patients with A + were further categorized into A + V + and A + V − groups, while CU participants with A − V − served as the control group. In addition, available neuropsychological tests and magnetic resonance imaging (MRI) data at 1-, 2-, and 4-year follow-ups were collected.

### Demographics and cognitive assessment

Demographic information was assessed, including age, sex, education level, and APOE ε4 status. Participants with one or more ε4 alleles were identified as APOE ε4 carriers. To address the potential confounding influence of APOE ε2/ε4, all analyses will be reexamined after excluding participants identified as APOE ε2/ε4 carriers (see Supplementary Material 1). Furthermore, vascular risk factors such as hypertension, hypercholesterolaemia, diabetes, and smoking status were evaluated.

All participants underwent comprehensive neuropsychological tests involving multiple cognitive domains, including memory (Auditory Verbal Learning Test [AVLT] total recall score for trials 1–5 and 30-min delayed recall), attention (Trail Making Test, Part A [TMT-A]), executive function (Trail Making Test, Part B [TMT-B]), and language (semantic verbal fluency [SVF]).

### MRI acquisition

All participants underwent whole‐brain MRI scans using 3.0 T scanners, according to ADNI protocol. The sequence parameters of T1-weighted inversion recovery spoiled gradient recalled images were as follows: repetition time (TR) = 6.96 ms, echo time (TE) = 2.8 ms, voxel size = 1.01 × 1.01 × 1.2 mm^3^, matrix size = 256 × 256, and flip angle = 11°. Diffusion tensor imaging (DTI) images were acquired using spin echo pulse sequence echo-planar-imaging (SE-EPI) with the following parameters: TR = 9000 ms, voxel size = 2.7 × 2.7 × 2.7 mm^3^, matrix size = 256 × 256, flip angle = 90°, and the number of slices = 59. Each DTI scan consists of 46 separate images: 5 T2-weighted images with no diffusion sensitization (b0 images) and 41 diffusion-weighted images (b = 1000 s/mm^2^). In addition, T2 FLAIR data were obtained only at baseline using an echo-planar imaging sequence with the following parameters: TR = 9000 ms, TE = 90 ms, TI = 2500 ms, number of slices = 42, and slice thickness = 5 mm.

### Cholinergic WM pathways analysis

The methods for tracking the cholinergic WM pathways largely followed the procedure described in previous studies [15, 31]. This approach comprised five key steps.

#### Preprocessing DTI data

We used MRtrix3 (http://www.mrtrix.org) to remove Gibbs ringing and correct for eddy-current, head motion, and bias field. Then, fiber-orientation distributions (FODs) were determined for each participant using Single-Shell, 3-Tissue Constrained Spherical Deconvolution (SS3T-CSD) [32]. The 3-tissue response functions were estimated directly from the diffusion MRI data itself and then averaged to obtain a group average anisotropic single-fiber WM response function and isotropic gray matter (GM) and CSF response functions using an unsupervised method [33]. Finally, bias field correction and intensity normalization in the log-domain were performed on the 3-tissue compartments.

#### Determination of regions of interest masks

Five regions of interest (ROIs) masks for cholinergic tractography—NBM, cingulum, external capsule, brainstem, and anterior commissure—were chosen based on the previous studies [15, 34]. These ROIs were then registered to individual diffusion space using a combination of nonlinear SyN registration algorithm [35] in Advanced Normalization Tools (ANTs, http://stnava.github.io/ANTs/) and FMRIB's Linear Image Registration Tool (FLIRT) [36].

#### Individual tractography

**Fig. 1:**
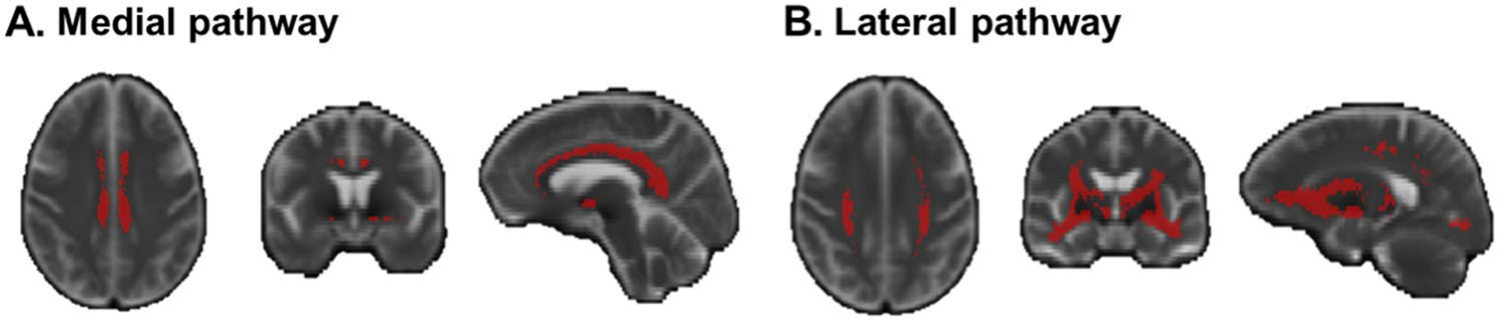
Cholinergic WM pathways. The masks of cholinergic medial pathway (**A**) and lateral pathway (**B**) were displayed, respectively

Tractography was performed on all CU participants using tckgen in MRtrix. The NBM ROI mask was designated as the seed mask, while the brainstem and anterior commissure ROI masks were set as exclusion mask. The cingulum and external capsule ROI masks were used to track cholinergic medial and lateral pathways, respectively. Tractography parameters: tractography algorithm: iFOD2; number of generated streamlines: 10,000; all other parameters, such as step size and angle constraints, were set to default values by MRtrix. Subsequently, a B0 template was created based on all CU participants’ B0 images using the ‘build-template’ module in ANTs. Individual cholinergic pathways were then registered to the B0 template space. Finally, only voxels that appeared in a minimum of 50% of the cases were preserved in template creation. The cholinergic WM pathways are shown in Fig. 1.

#### Individual cholinergic pathway

After acquiring the cholinergic pathway templates, the individual medial and lateral pathways were warped into their respective individual spaces. Manual inspection was conducted to ensure their accuracy in individual space.

#### Cholinergic pathway integrity evaluation

The average MD index, which has previously been demonstrated to be sensitive to injury of cholinergic pathways [15–17], was utilized to characterize the microstructural properties of the cholinergic WM pathways.

### NBM volumes

The methodology for obtaining the NBM volume involved three primary steps.

#### T1 image segmentation

Utilizing the Longitudinal segmentation pipeline in the Computational Anatomy Toolbox (CAT12, http://dbm.neuro.unijena.de/cat/), all T1 images across different time points underwent preprocessing. Specifically, settings optimized for detecting significant changes such as aging or developmental effects were selected within the Longitudinal model option. Modulated GM/WM segmentations were chosen to compensate for spatial normalization effects. Initially, rigid alignment of individual T1 images created an average image, subsequently registered to CAT12's default-defined standard space. Within this standard space, subject-specific tissue probability maps for GM and WM were generated for each time point. Total intracranial volume (TIV) was also obtained from this segmentation process.

#### NBM mask

NBM mask, initially acquired in MNI space, was registered to CAT12's default-defined standard space. This registration process involved nonlinear SyN registration algorithm in ANTs to align the MNI space T1 images with CAT12’s default-defined standard space T1 images. The deformation map obtained from this alignment was then applied to the NBM mask, resulting in the acquisition of CAT12’s default-defined standard space NBM mask.

#### NBM volume calculation

The GM probability map obtained in the first step and the NBM mask acquired in the second step were multiplied together to calculate the NBM volume for each participant.

### Measurement of WMH volume

Quantitative WMH volumes were also measured on baseline T2 FLAIR images by an automatic segmentation tool (Lesion Segmentation Tool, LST) using a lesion prediction algorithm (LPA) based on Statistical Parametric Mapping software (SPM12, http://www.fil.ion.ucl.ac.uk/spm). The automatically created WMH images were then manually corrected to avoid incorrect segmentation. WMH volumes were automatically extracted by LST. For analysis, WMH volumes were normalized to the TIV and subsequently log-transformed to meet normal distribution.

### Statistical analysis

Statistical analysis was performed using SPSS statistical software (version 26; SPSS, Inc., Chicago, IL) and R studio (version 4.1.3). Results were considered statistically significant at *P* < 0.05 (two-tailed).

#### Cross-sectional comparison among groups

Age, years of education, cognitive measures, cerebral Aβ levels, and WMH volume were compared among the CU_A−V−_, MCI_A+V−_, and MCI_A+V+_ groups using one-way analysis of variance (ANOVA), followed by post hoc tests with Bonferroni correction for multiple comparisons. The Chi-square test was used for categorical variables, including sex, APOE genotype, and vascular risk factors. After adjusting for age, sex, education, and TIV, the NBM volume and cholinergic pathways MD were compared between groups using ANOVA, followed by pairwise post hoc tests with Bonferroni correction.

#### Cross-sectional associations between WMH burden and cholinergic deficits and the mediation analysis in MCI patients

We conducted partial correlation analysis to examine the relationship between WMH burden and cholinergic deficits in MCI patients, with age, sex, and education as covariates. To investigate the potential mediating role of cholinergic deficits in the connection between WMH burden and cognitive impairment, we initially conducted partial correlation analyses involving the cholinergic system and cognitive performance. This step was essential as mediation analysis requires the presence of significant associations between WMH burden and the cholinergic system, as well as between the cholinergic system and cognitive performance. Subsequently, upon confirming these significant associations, we employed mediation analysis to determine whether WMH could potentially contribute to cognitive impairment through cholinergic injury in MCI patients using the PROCESS macro v3.5 in SPSS. We performed bias-corrected bootstrapping with 5000 replications to estimate the indirect effect. An indirect effect through mediators between the independent and dependent variables is significant if the 95% confidence interval (CI) does not include zero. In this analysis, we considered normalized NBM volume (TIV-corrected), medial pathway MD, and lateral pathway MD as separate mediators, with WMH volume as the independent variable. Different cognitive domains were successively entered as dependent variables. Age, sex, and education were included as covariates.

#### Longitudinal changes in cholinergic system among groups

Linear mixed models were used to examine longitudinal changes in cholinergic system. The analyses were performed using the ‘lme4’ package R studio [37, 38]. We tested the longitudinal changes in cholinergic system among groups. The model included age, sex, education, group (CU_A−V−_ vs. MCI_A+V−_, CU_A−V−_ vs. MCI_A+V+_, MCI_A+V−_ vs. MCI_A+V+_), time (i.e., number of years from baseline), and group × time as fixed effects, while time was modeled as a random effect (random intercepts and slopes) for each participant. We separately analyzed three dependent cholinergic markers including normalized NBM volume, medial pathway MD, and lateral pathway MD.

#### Mediating effects of cholinergic changes between WMH burden and cognitive changes in MCI patients

To explore the potential mediating role of changes in the cholinergic system in the relationship between WMH burden and cognitive alterations, we conducted partial correlation analyses involving WMH burden and cholinergic changes, as well as cholinergic changes and cognitive changes. After confirming these significant associations, we used mediation analysis to study whether WMH burden contribute to cognitive changes by accelerating cholinergic system degeneration. Baseline WMH burden was considered as independent variable. Slopes of normalized NBM volume change, medial pathway MD and lateral pathway MD were set as mediators separately. Different cognitive domains slopes were successively entered as dependent variables. The measurement of slopes was extracted for each patient using linear mixed-effects regression with random effects of intercept and linear slope (with respect to time). Age, sex, and education were included as covariates.

## Results

### Demographic and clinical data

A total of 40 CU_A−V−_ participants, 29 MCI_A+V−_ patients, and 23 MCI_A+V+_ patients were included. The demographic characteristics, cognitive performance, cerebral Aβ levels, and WMH volume at baseline were summarized in Table 1. The MCI_A+V+_ group (77.61 ± 4.70) was significantly older than the CU_A−V−_ group (71.85 ± 6.10, *P* = 0.004) and MCI_A+V−_ group (72.36 ± 8.25, *P* = 0.016). There were no significant differences in sex and education level among groups. The frequency of APOE ε4 carriers was significantly higher in both MCI groups in comparison to the CU_A−V−_ group. Hypertension was more prevalent in the MCI_A+V+_ group compared to the MCI_A+V−_ group, while there were no significant differences for other risk factors. Both MCI groups had lower scores than the CU_A−V−_ group in MMSE (MCI_A+V−_: *P* < 0.001, MCI_A+V+_:* P* = 0.041), AVLT trials 1–5 (MCI_A+V−_: *P* < 0.001, MCI_A+V+_: *P* < 0.001), AVLT delayed recall (MCI_A+V−_: *P* < 0.001, MCI_A+V+_: *P* < 0.001), and SVF (MCI_A+V−_: *P* = 0.003, MCI_A+V+_: *P* = 0.004). Furthermore, the MCI_A+V+_ group had lower scores than the MCI_A+V−_ and CU_A−V−_ groups in TMT-A (MCI_A+V−_: *P* = 0.005, CU_A−V−_: *P* < 0.001) and TMT-B (MCI_A+V−_: *P* = 0.009, CU_A−V−_: *P* < 0.001).

**Table 1:** Demographics and clinical characteristics at baseline

Characteristics	CU_A−V−_ (*n* = 40)	MCI_A+V−_ (*n* = 29)	MCI_A+V+_ (*n* = 23)	*F*-value/χ^2^-value	*P*-value
Demographics					
Age, years	71.85 (6.10)	72.36 (8.25)	77.61 (4.70)^ab^	6.21	0.003
Sex (F/M)	19/21	14/15	6/17	3.34	0.188
Education, years	16.75 (2.64)	15.17 (2.55)	15.74 (3.12)	2.93	0.059
APOE ε4 carriers, *n* (%)	10 (25%)	21 (72.4%)^a^	16 (69.6%)^a^	19.32	< 0.001
Vascular risk factors, *n* (%)					
Hypertension	21 (52.5%)	13 (44.8%)	18 (78.3%)^b^	6.30	0.043
Hypercholesterolaemia	18 (45.0%)	11 (37.9%)	13 (56.5%)	1.80	0.407
Diabetes	6 (15.0%)	1 (3.4%)	6 (26.1%)	5.46	0.065
Current or past smoking	6 (15.0%)	4 (13.8%)	5 (21.7%)	0.68	0.711
Cognitive performance					
MMSE	28.78 (1.67)	27.07 (1.85)^a^	27.65 (1.56)^a^	8.93	< 0.001
AVLT trials 1–5	48.40 (11.11)	30.48 (7.61)^a^	31.13 (8.87)^a^	38.357	< 0.001
AVLT delayed recall	8.15 (4.28)	2.31 (2.70)^a^	3.61 (3.10)^a^	25.518	< 0.001
Log-transformed TMT-A	1.49 (0.17)	1.54 (0.12)	1.69 (0.17)^ab^	11.46	< 0.001
Log-transformed TMT-B	1.86 (0.18)	1.95 (0.21)	2.12 (0.21)^ab^	12.53	< 0.001
SVF (animal)	20.60 (5.42)	16.24 (4.32)^a^	16.04 (5.81)^a^	8.26	< 0.001
Neuroimaging					
Cerebral Aβ levels	1.02 (0.05)	1.38 (0.17)^a^	1.44 (0.16)^a^	105.02	< 0.001
Log-transformed WMH volume (TIV corrected)	− 2.76 (0.40)	− 2.65 (0.30)	− 2.08 (0.21)^ab^	32.11	< 0.001

Values are expressed as mean (standard deviation), number of participants

*Aβ *amyloid beta; *AVLT* auditory verbal learning test; *CU* cognitively unimpaired; *MCI* mild cognitive impairment; *MMSE* mini-mental state examination; *SVF* semantic verbal fluency; *TIV* total intracranial volume; *TMT* trail making test; *WMH* white matter hyperintensities

^a^Compared to CU_A−V−_ group, *P* < 0.05

^b^Compared to MCI_A+V−_ group, *P* < 0.05

In addition, detailed information on the longitudinal changes of cognitive performance among groups can be found in Supplementary Material 2. Briefly, both MCI groups exhibited an accelerated longitudinal decline on most cognitive measures compared to the CU_A−V−_ group. Moreover, the MCI_A+V+_ group further showed faster cognitive decline in TMT-B than the MCI_A+V−_ group.

### Comparison of baseline NBM volume and WM pathways MD among groups

**Fig. 2:**
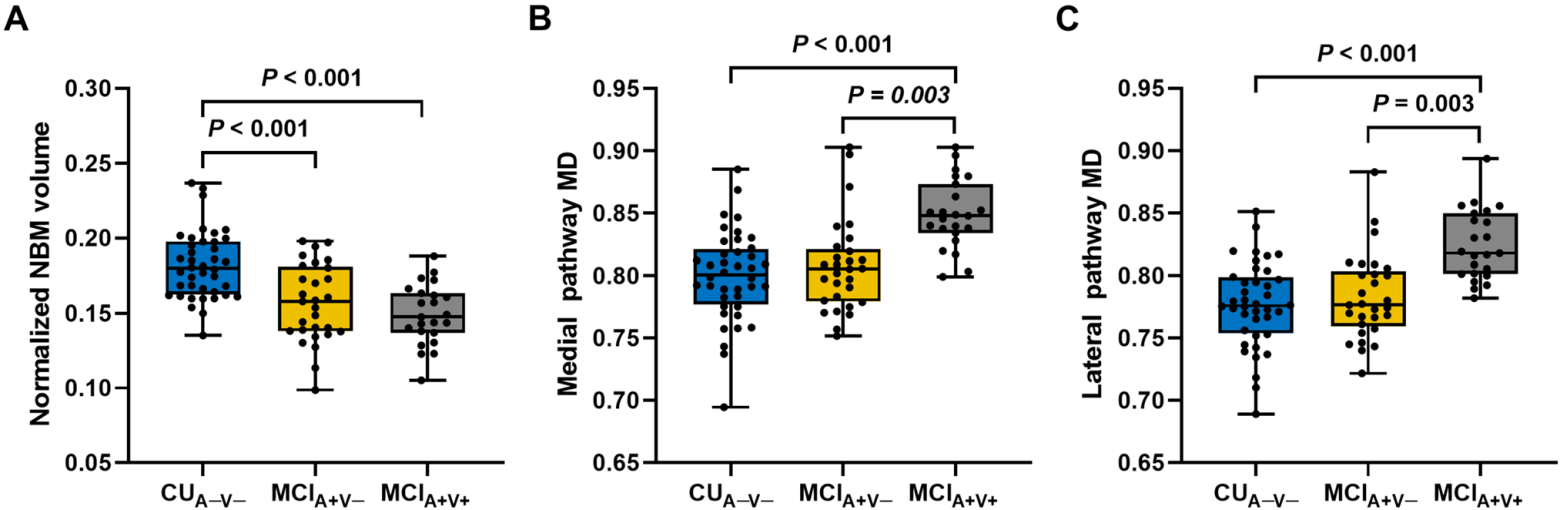
Comparison of baseline integrity of NBM and WM pathways among groups. The normalized NBM volume (**A**), medial pathway MD (**B**), and lateral pathway MD (**C**) were compared between CU_A−V−_, MCI_A+V−_, and MCI_A+V+_ groups. *P*-values result from post hoc tests with Bonferroni correction for multiple comparisons. *P*-values for all comparisons not shown are > 0.05. *CU* cognitively unimpaired; *MCI* mild cognitive impairment; *MD* mean diffusivity; *NBM* nucleus basalis of Meynert

After adjusting for age, sex, education, and TIV, both the MCI_A+V+_ and MCI_A+V−_ groups showed reduced NBM volume compared to the CU_A−V−_ group at baseline (*P* < 0.001 for both MCI groups), However, there was no significant difference in NBM volume between the two MCI groups (Fig. 2A).

The MCI_A+V+_ group showed increased MD in the medial and lateral pathways compared to the MCI_A+V−_ group (both pathways: *P* = 0.003) and CU_A−V−_ group (both pathways: *P* < 0.001) after adjusting for age, sex, education, and TIV. There was no significant difference in MD of the two pathways between the MCI_A+V−_ and CU_A−V−_ groups (Fig. 2B, C).

### Baseline associations between WMH burden and cholinergic markers as well as mediation analysis in MCI patients

**Fig. 3:**
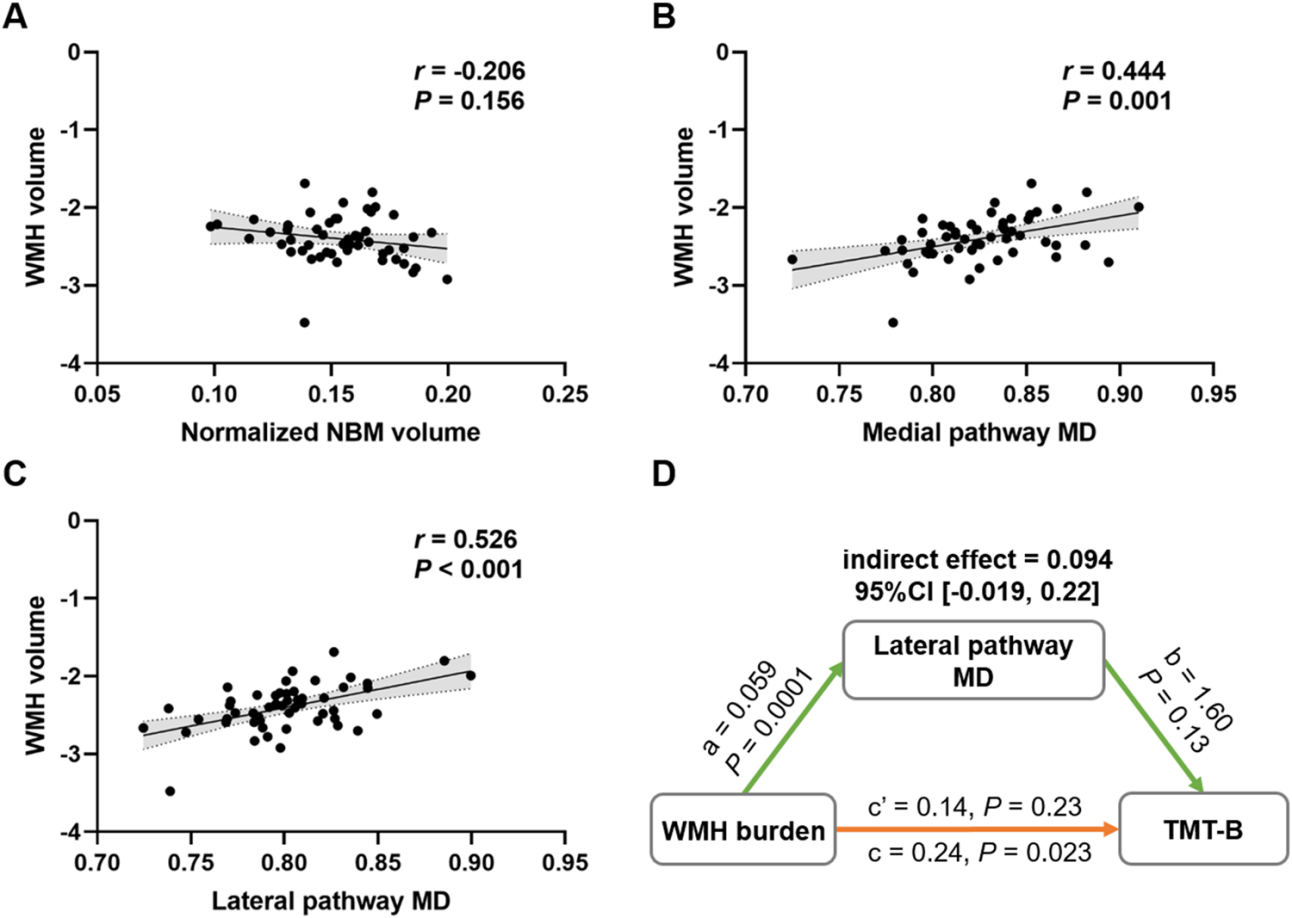
Baseline associations between WMH burden and cholinergic markers as well as mediation analysis in MCI patients. Scatter plots graphs of relationship between: WMH volume and normalized NBM volume (**A**); WMH volume and medial pathway MD (**B**); WMH volume and lateral pathway MD (**C**) in MCI patients. Mediation analysis showed that lateral pathway MD did not mediate the relationship between WMH burden and TMT-B (**D**). *a* = regression coefficient for WMH burden and lateral pathway MD; *b* = regression coefficient for lateral pathway MD and TMT-B, adjusted for the effect of WMH burden; *c*′ = the direct effect for WMH burden and TMT-B; *c* = the total effect. An indirect effect is significant if the 95% CI does not include zero. *CI* confidence interval; *MD*  mean diffusivity; *NBM* nucleus basalis of Meynert; *TMT-B* Trail making test, part B; *WMH*  white matter hyperintensities

As shown in Fig. 3, WMH volume had significant associations with MD in cholinergic pathways (medial pathway: *r *= 0.444, *P* = 0.001; lateral pathway: *r* = 0.526, *P* < 0.001), but not with normalized NBM volume (*r* =  − 0.206, *P* = 0.156). The correlations between cholinergic deficits and cognitive impairment were present in Supplementary Material 3. In the mediation analysis, the lateral pathway MD did not act as a mediator for the impact of WMH burden on TMT-B (indirect effect = 0.094, 95%CI [−0.019, 0.22]), although the total effect of WMH burden on TMT-B was significant (total effect = 0.24, *P* = 0.023).

### Longitudinal changes in NBM volume and WM pathways MD among groups

**Fig. 4:**
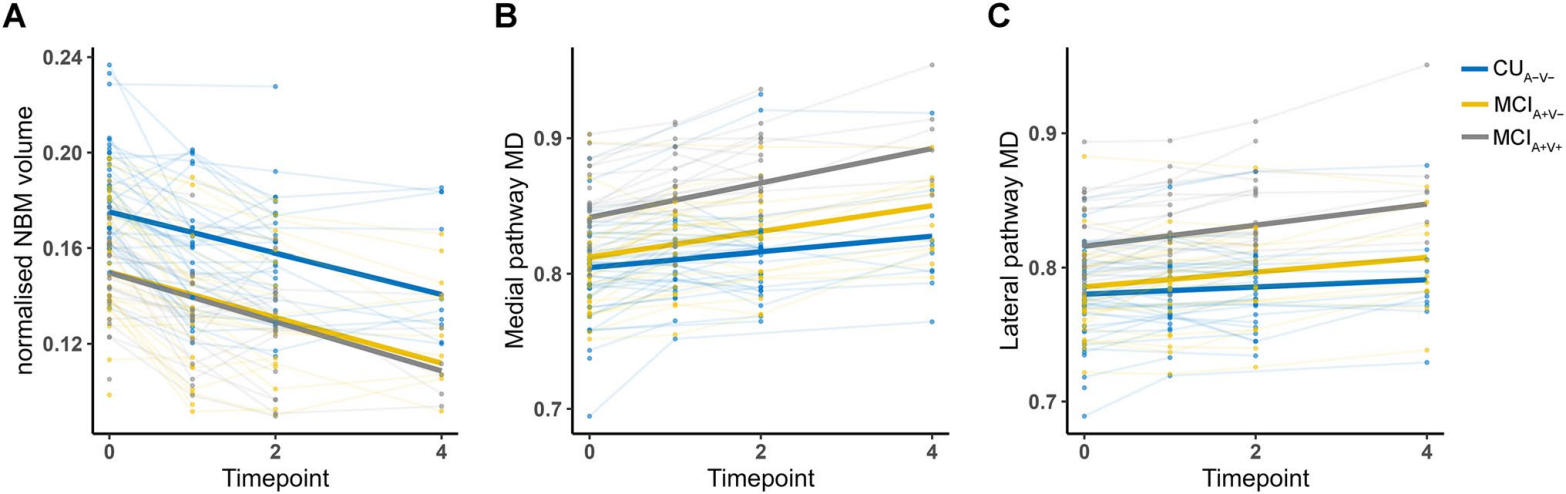
Longitudinal changes of NBM volume and WM pathways integrity. The longitudinal evolutionary trajectories of cholinergic markers in the CU_A−V−_ (blue), MCI_A+V−_ (yellow), and MCI_A+V+_ (gray) groups (**A**–**C**). The thin lines represent the changes in individual cholinergic markers over time, and the corresponding thick lines represent the estimated average cholinergic markers changes of the three groups. The timepoint on the *X*-axis refers to the year. *CU* cognitively unimpaired; *MCI* mild cognitive impairment; *MD* mean diffusivity; *NBM* nucleus basalis of Meynert

Normalized NBM volume decreased over time in the three groups (*P* < 0.001). However, there was no significant difference in the rate of normalized NBM volume change between the MCI_A+V+_ and CU_A−V−_ groups (*P* = 0.141), the MCI_A+V−_ and CU_A−V−_ groups (*P* = 0.141), and the MCI_A+V+_ and MCI_A+V−_ groups (*P* = 0.921) (Fig. 4A).

Both MCI groups exhibited an increase in MD of medial and lateral pathways over time (both pathways: *P* < 0.001). The MCI_A+V+_ group further displayed a faster longitudinal MD increase in both medial and lateral pathways compared to CU_A−V−_ group (medial pathway: *P* = 0.003; lateral pathway: *P* = 0.038). In addition, the MCI_A+ V−_ group also showed faster longitudinal change in medial pathway MD compared to CU_A−V−_ group (*P* = 0.024). However, we did not find significant difference in longitudinal change in lateral pathway MD between the MCI_A+V−_ and CU_A−V−_ groups (*P* = 0.085), or bilateral pathways MD between the MCI_A+V+_ and MCI_A+V−_ groups (medial pathway: *P* = 0.151; lateral pathway: *P* = 0.316) (Fig. 4B, C). Further details are available in Supplementary Material 4.

### Mediation analysis of longitudinal cholinergic system changes between WMH burden and cognitive changes in MCI patients

**Fig. 5:**
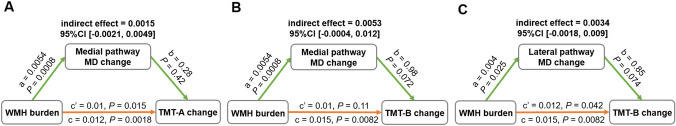
Mediation analysis of longitudinal cholinergic changes between WMH burden and cognitive changes in MCI patients. Mediation analysis showed that the change in medial pathway MD did not mediate the relationship between WMH burden and changes in TMT-A (**A**) and TMT-B (**B**). In addition, the change in lateral pathway MD also did not mediate the relationship between WMH burden and change in TMT-B (**C**). *a* = regression coefficient for WMH burden and cholinergic pathway MD change; *b* = regression coefficient for cholinergic pathway MD change and cognitive change, adjusted for the effect of WMH burden; *c*′ = the direct effect for WMH burden and cognitive change; *c* = the total effect. An indirect effect is significant if the 95% CI does not include zero. *MD *mean diffusivity; *TMT-A* trail making test, part A; *TMT-B*  trail making test, part B; *WMH*  white matter hyperintensities

The detailed relationships between WMH burden and longitudinal changes in cholinergic markers and cognitive performance were present in Supplementary Material 5. In the mediation analysis, the change in medial pathway MD did not act as a mediator for the impact of WMH burden on the changes observed in TMT-A (indirect effect = 0.0015, 95%CI [−0.0021, 0.0049], Fig. 5A) and TMT-B (indirect effect = 0.0053, 95%CI [−0.0004, 0.012], Fig. 5B). Furthermore, the change in lateral pathway MD also did not serve as a mediator for the effect of WMH burden on the change observed in TMT-B (indirect effect = 0.0034, 95%CI [−0.0018, 0.009], Fig. 5C). However, we found that WMH burden was significantly correlated with the rate of change in both cholinergic pathways. Besides, WMH burden had significant total or direct effects on the longitudinal change in TMT-A and TMT-B. See details in Fig. 5.

## Discussion

We investigated the effect of SVD on cholinergic system in MCI patients and its contribution to cognitive impairment through cholinergic degeneration. Our main findings are (1) MCI patients with SVD showed disrupted integrity in cholinergic medial and lateral pathways compared to those without SVD and CU participants. Furthermore, SVD burden was associated with the disruption of cholinergic pathways across all MCI patients. (2) Longitudinally, SVD burden could accelerate the degeneration of cholinergic pathways. (3) However, we found no differences in baseline NBM volume or its rate of change over time between MCI patients with and without SVD. (4) Additionally, our study, by cross-sectional and longitudinal mediation analysis, did not support the role of SVD in cognitive impairment through cholinergic system degeneration.

Our findings revealed that MCI patients with SVD had reduced integrity of both cholinergic pathways at baseline compared to those without SVD and CU participants. Moreover, their decline in pathway integrity over time was faster than that of CU participants. These results suggest that SVD not only disrupts the integrity of cholinergic pathways in MCI patients, but also accelerates cholinergic degeneration over time. Damaged integrity of cholinergic pathways has been observed in subcortical vascular cognitive impairment [39], which supports our results. Moreover, our cross-sectional and longitudinal correlation analyses showed significant associations between increased WMH burden and cholinergic pathway disruption. One possible explanation is that WM lesions may directly destroy the integrity of cholinergic projections, which are mostly unmyelinated, and therefore susceptible to WM damage [14]. Our findings align with prior research reporting a negative association between WMH burden and cholinergic pathways integrity in both cognitively normal individuals [15] and patients with vascular cognitive impairment [39]. Moreover, several studies have indicated that degradation of the cholinergic projections caused by WMH may contributed to cognitive dysfunction in AD [24, 40]. Interestingly, Cedres et al. [41] further demonstrated that WMH burden is a more critical factor than AD pathologies such as Aβ42/40 ratio and phosphorylated tau levels in CSF in contributing to the degeneration of cholinergic pathways in cognitively unimpaired individuals. Together, these findings emphasize the role of SVD in the degeneration of cholinergic WM pathways.

In addition, we observed reduced NBM volume in both MCI groups compared to the CU group at baseline. Cholinergic degeneration, which includes neuronal loss [9, 10] to morphological changes in the NBM, has been extensively documented in patients with MCI, AD [11, 34, 42] and mixed AD and vascular pathologies [43], which supports our findings. However, our study did not find a significant difference in baseline NBM volume between MCI patients with and without SVD or in its rate of change over time. Our correlation analysis also demonstrated no association between NBM volume and WMH burden. Previous studies have documented that NBM volume is not reduced in patients with vascular cognitive impairment when compared to healthy elderly participants [39, 43]. More recently, Kindler et al. further reported no significant correlation between CHIPS score and NBM volume in AD patients [27]. However, only a few studies have shown significant association between WMH burden and NBM volume in individuals with normal cognition [15] and subtle cognitive impairment [31], which could be attributed to the retrograde degeneration of the NBM from WMH strategically damaging cholinergic projections [44]. A possible explanation for the discrepancy could be that the significant association between WMH burden and NBM degeneration may be stronger in normal cognition and subtle cognitive impairment but weaken or disappear when apparent cognitive impairment occurs.

The mediation analyses showed significant total or direct effects of SVD burden on attention and executive functions across all MCI patients. Our data are in line with previous studies that reported significant associations between SVD and cognitive decline, particularly in the attention and executive functions [45, 46]. Unexpectedly, we did not find an indirect effect of cholinergic pathway deficits between SVD burden and cognitive impairment. A possible explanation could due to the small sample size in our study which make it difficult to capture the mediating role of cholinergic pathway between SVD and cognitive changes. Furthermore, it is likely that the participants in our study were highly educated. Previous research has shown that a higher level of education represents greater cognitive reserve, which may attenuate the negative impact of SVD on cognition [47]. Additionally, cholinergic activity can be upregulated in MCI patients with a higher education level, which appears to have a compensatory effect [48, 49]. Therefore, they may have influenced our exploration of the relationship between SVD, cholinergic system integrity, and cognitive changes.

There are several limitations of our study that should be noted. Firstly, participants in the ADNI database are highly educated, which may introduce selection bias and limit the generalizability of our findings. In addition, the relatively small sample size was constrained by the requirement for complete clinical, MRI, and PET data. Therefore, repeating this work in a population-based cohort with a larger sample size may provide better insight into the impact of SVD on cholinergic system in AD. Secondly, participants were not age-matched, with MCI patients with SVD being older than other groups. However, we accounted for the age effect in our analyses. Thirdly, we used WMH as the SVD index to explore the influence of SVD on cholinergic system. Notably, other components of SVD, such as lacunes, microbleeds, and perivascular spaces, may have different etiologies. Future studies could assess the contribution of a composite SVD index or other components to cholinergic degeneration. Finally, despite employing a longitudinal design, the follow-up period was limited to 48 months. Future studies with extended follow-up durations are required to explore the influence of SVD on cholinergic system changes in AD.

## Conclusions

In summary, our findings provide important evidence for the influence of SVD on cholinergic system disruptions and highlight the value of WM pathways microstructure in revealing cholinergic deficits in MCI patients with mixed amyloid and vascular pathologies. Future studies require large sample sizes and population-based cohorts to explore the role of SVD on cognitive impairment through cholinergic system degeneration in AD.

### Supplementary Information

Below is the link to the electronic supplementary material.

Supplementary file1 (DOCX 1046 KB)

## Data Availability

Data used in preparation of this article were obtained from the Alzheimer’s disease Neuroimaging Initiative (ADNI) database (http://www.loni.usc.edu). As such, the investigators within the ADNI contributed to the design and implementation of ADNI and/or provided data but did not participate in analysis or writing of this report. A complete listing of ADNI investigators can be found at: http://adni.loni.usc.edu/wpcontent/uploads/how_to_apply/ADNI_Acknowledgement_List.pdf.

## Abbreviations

Aβ: Amyloid beta
AChE: Acetylcholinesterase
AD: Alzheimer’s disease
ADNI: Alzheimer’s disease neuroimaging initiative
ANOVA: One-way analysis of variance
ANTs: Advanced normalization tools
AVLT: Auditory verbal learning test
BF: Basal forebrain
CAT: Computational anatomy toolbox
CDR: Clinical dementia rating
CHIPS: Cholinergic pathways hyperintensities scale
CSF: Cerebrospinal fluid
CU: Cognitively unimpaired
DTI: Diffusion tensor imaging
FLAIR: Fluid‐attenuated inversion recovery
FLIRT: FMRIB's linear image registration tool
FODs: Fiber-orientation distributions
GM: Gray matter
LST: Lesion segmentation tool
LPA: Lesion prediction algorithm
MCI: Mild cognitive impairment
MD: Mean diffusivity
MMSE: Mini-mental state examination
MRI: Magnetic resonance imaging
NBM: Nucleus basalis of Meynert
PET: Positron emission tomography
ROIs: Regions of interest
SE-EPI: Spin echo pulse sequence echo-planar-imaging
SPM: Statistical parametric mapping
SS3T-CSD: Single-shell, 3-tissue constrained spherical deconvolution
SUVR: Standardized uptake value ratio
SVD: Small vessel disease
SVF: Semantic verbal fluency
TE: Echo time
TIV: Total intracranial volume
TMT: Trail making test
TR: Repetition time
WM: White matter
WMH: White matter hyperintensities
