# Supplementary material for: Effect of cerebral small vessel disease on the integrity of cholinergic system in mild cognitive impairment patients: a longitudinal study
Source: J Neurol. 2024 Feb 21;271(5):2704–15. doi: 10.1007/s00415-024-12218-2 (PMC11055699; doi:10.1007/s00415-024-12218-2)
Supplement: Supplementary file 1 — Supplementary file1 (DOCX 1046 KB) [file 415_2024_12218_MOESM1_ESM.docx]

**Supplementary Materials**

**Supplementary Material 1**

**APOE genotyping**

APOE genotype was determined through genotyping the two single-nucleotide polymorphisms (rs429358, rs7412) that define the APOE-ε2, ε3, and ε4 alleles. DNA extraction was performed by genics from a 3-mL aliquot of EDTA blood (adni.loni.usc.edu/data-samples/genetic-data/).

| **Table S1. APOE genotypes of the three groups.** | | | |
| --- | --- | --- | --- |
| **Genotype** | **CU_A−V−_  (n=40)** | **MCI_A+V−_ (n=29)** | **MCI_A+V+_  (n=23)** |
| ε2/ε3, n | 4 | 0 | 1 |
| ε3/ε3, n | 26 | 8 | 6 |
| ε2/ε4, n | 0 | 0 | 1 |
| ε3/ε4, n | 10 | 13 | 13 |
| ε4/ε4, n | 0 | 8 | 2 |
|  |  |  |  |

Upon considering the potential distinct impact of APOE ε2/ε4 status on the integrity of the cholinergic system compared to other ε4 combinations (such as ε3/ε4 and ε4/ε4), we conducted a reanalysis of our findings. Specifically, we reexamined our results after the exclusion of one participant identified as an APOE ε2/ε4 carrier within the MCI_A+V+_ group. Upon careful reevaluation, the results obtained after removing the APOE ε2/ε4 carrier align consistently with the original findings detailed in the manuscript. A comprehensive breakdown of these revised results is shown below:

***Demographic and clinical data***

| **Table S2. Demographics and clinical characteristics at baseline.** | | | | | |
| --- | --- | --- | --- | --- | --- |
| **Characteristics** | **CU_A−V−_  (n=40)** | **MCI_A+V−_ (n=29)** | **MCI_A+V+_  (n=22)** | **F-value/ χ2-value** | ***P*-value** |
| **Demographics** |  |  |  |  |  |
| Age, years | 71.85(6.10) | 72.36(8.25) | 77.26(4.49)^ab^ | 5.30 | 0.007 |
| Sex (F/M) | 19/21 | 14/15 | 6/16 | 2.88 | 0.237 |
| Education, years | 16.75(2.64) | 15.17(2.55) | 15.91(3.09) | 2.85 | 0.063 |
| APOE ε4 carriers, n (%) | 10(25%) | 21(72.4%)^a^ | 15(68.2%)^a^ | 18.73 | < 0.001 |
| **Vascular risk factors, n (%)** |  |  |  |  |  |
| Hypertension | 21(52.5%) | 13(44.8%) | 17(77.3%) | 5.71 | 0.058 |
| Hypercholesterolaemia | 18(45.0%) | 11(37.9%) | 12(54.5%) | 1.40 | 0.498 |
| Diabetes | 6(15.0%) | 1(3.4%) | 5(22.7%) | 4.27 | 0.118 |
| Current or past smoking | 6(15.0%) | 4(13.8%) | 5(22.7%) | 0.84 | 0.657 |
| **Cognitive performance** |  |  |  |  |  |
| MMSE | 28.78(1.67) | 27.07(1.85)^a^ | 27.73(1.55) | 8.75 | < 0.001 |
| AVLT trials 1–5 | 48.40(11.11) | 30.48(7.61)^a^ | 31.00(9.06)^a^ | 37.91 | < 0.001 |
| AVLT delayed recall | 8.15(4.28) | 2.31(2.70)^a^ | 3.59(3.17)^a^ | 25.19 | < 0.001 |
| Log-transformed TMT-A | 1.49(0.17) | 1.54(0.12) | 1.68(0.17)^ab^ | 10.24 | < 0.001 |
| Log-transformed TMT-B | 1.86(0.18) | 1.95(0.21) | 2.10(0.20)^ab^ | 11.00 | < 0.001 |
| SVF (animal) | 20.60(5.42) | 16.24(4.32)^a^ | 16.27(5.84)^a^ | 7.81 | < 0.001 |
| **Neuroimaging** |  |  |  |  | < 0.001 |
| Cerebral Aβ levels | 1.02(0.05) | 1.38(0.17)^a^ | 1.44(0.16)^a^ | 106.30 | < 0.001 |
| Log-transformed WMH volume (TIV corrected) | −2.76(0.40) | −2.65(0.30) | −2.08(0.21)^ab^ | 30.88 | < 0.001 |
| Note: Values are expressed as mean (standard deviation), number of participants. Abbreviations: Aβ = amyloid beta; AVLT = Auditory Verbal Learning Test; CU = cognitively unimpaired; MCI = mild cognitive impairment; MMSE = Mini-Mental State Examination; SVF = Semantic Verbal Fluency; TIV = total intracranial volume; TMT = Trail Making Test; WMH = white matter hyperintensities. ^a^ compared to CU_A−V−_ group, *P* < 0.05. ^b^ compared to MCI_A+V−_ group, *P* < 0.05. | | | | | |

After excluding one participant with APOE ε2/ε4 in the MCI_A+V+_ group, the demographics and clinical characteristics at baseline among the three groups are generally consistent with the original results.

***Figure S1. Comparison of baseline NBM volume and WM pathways MD among groups***

After excluding one participant with APOE ε2/ε4 in the MCI_A+V+_ group, the MCI_A+V+_ group also showed reduced NBM volume compared to the CU_A−V−_ group at baseline (*P* < 0.001). Moreover, the MCI_A+V+_ group also showed increased MD in the medial and lateral pathways compared to the MCI_A+V−_ group (medial pathway: *P* = 0.002, lateral pathway: *P* = 0.004) and CU_A−V−_ group (both pathways: *P* < 0.001).


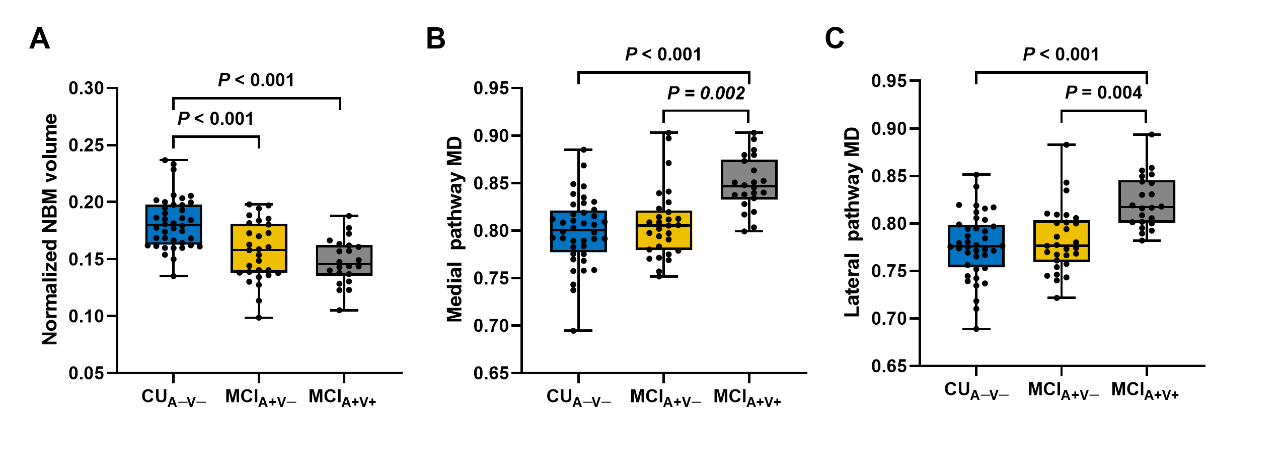


***Figure S2. Longitudinal changes of NBM volume and WM pathways MD among groups***

After excluding one participant with APOE ε2/ε4 in the MCI_A+V+_ group, there was also no significant difference in the rate of normalized NBM volume change between the MCI_A+V+_ and CU_A−V−_ groups (*P* = 0.352), the MCI_A+V−_ and CU_A−V−_ groups (*P* = 0.352), and the MCI_A+V+_ and MCI_A+V−_ groups (*P* = 0.834).

The MCI_A+V+_ group displayed a faster longitudinal MD increase in both medial and lateral pathways compared to CU_A−V−_ group (medial pathway: *P* = 0.003; lateral pathway: *P* = 0.033). In addition, the MCI_A+ V−_ group also showed faster longitudinal change in medial pathway MD compared to CU_A−V−_ group (*P* = 0.019). However, we did not find significant difference in longitudinal change of lateral pathway MD between the MCI_A+V−_ and CU_A−V−_ groups (*P* = 0.067), or bilateral pathways MD between the MCI_A+V+_ and MCI_A+V−_ groups (medial pathway: *P* = 0.164; lateral pathway: *P* = 0.348).


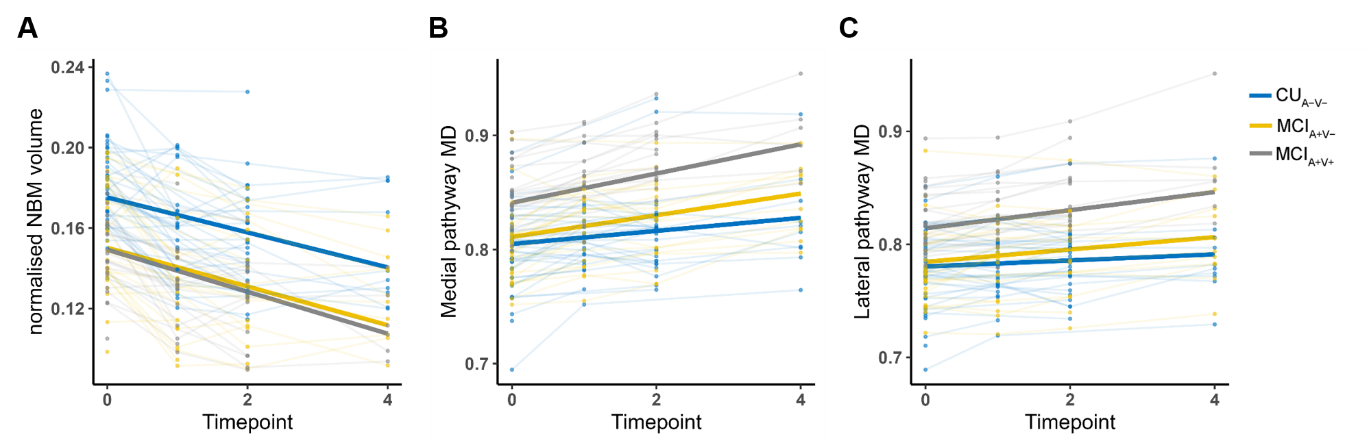


***Figure S3. Baseline associations between WMH burden and cholinergic markers as well as mediation analysis in MCI patients***

After excluding one participant with APOE ε2/ε4 in the MCI_A+V+_ group, WMH volume also had significant associations with MD in cholinergic pathways (medial pathway: r = 0.444, *P* = 0.002; lateral pathway: r = 0.539, *P* < 0.001), but not with normalized NBM volume (r = −0.198, *P* = 0.177). In addition, mediation analysis showed that lateral pathway MD also did not mediate the effect of WMH burden on TMT-B (indirect effect = 0.075, 95%CI [-0.037, 0.20]).

***
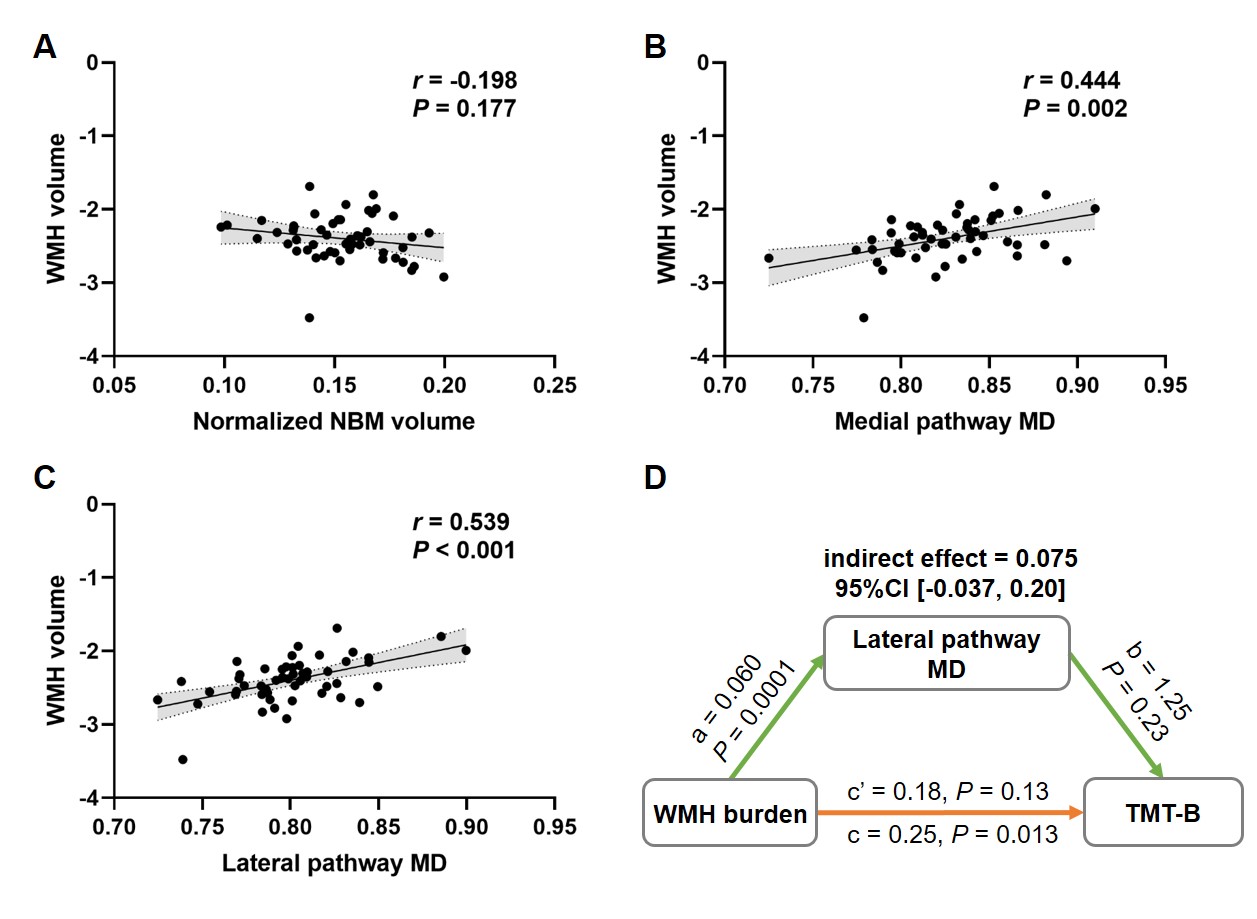
***

***Figure S4. Mediation analysis of longitudinal cholinergic system changes between WMH burden and cognitive changes in MCI patients***

After excluding one participant with APOE ε2/ε4 in the MCI_A+V+_ group, the change in medial pathway MD also did not mediate the effect of WMH burden on change in TMT-A (indirect effect = 0.0015, 95%CI [-0.0021, 0.0049]) and TMT-B (indirect effect = 0.0053, 95%CI [-0.0008, 0.012]). In addition, the change in lateral pathway MD also did not mediate the effect of WMH burden on change in TMT-B (indirect effect = 0.0034, 95%CI [-0.0015, 0.0088]).

***
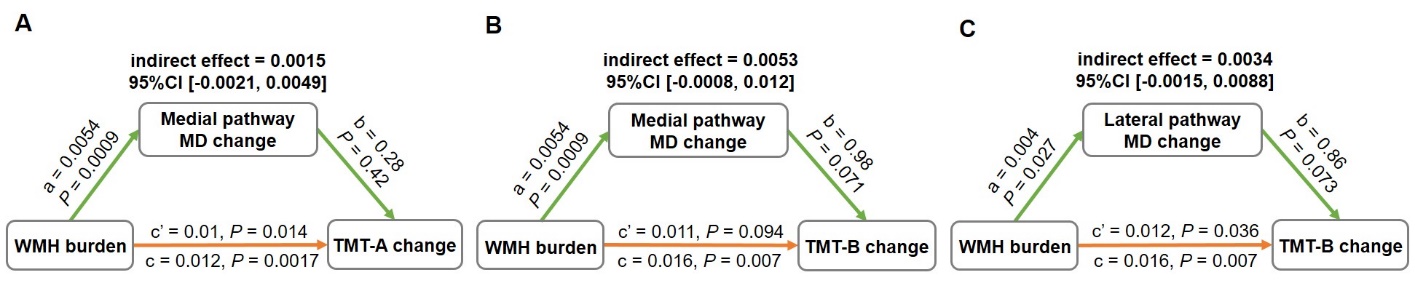
***

| **Supplementary Material 2**  **Table S3. Longitudinal changes of cognitive performance among groups.** | | | | |  |
| --- | --- | --- | --- | --- | --- |
| **Variables**^*^ | **CU_A−V−_** | **MCI_A+V−_** | **MCI_A+V+_** | ***P*-value** | |
| **MMSE** |  |  |  | *P*(CU_A−V−,_  MCI_A+V−_) < 0.001 | |
| Baseline | 28.78(1.67) | 27.07(1.85) | 27.65(1.56) | *P*(CU_A−V−_, MCI_A+V+_) < 0.0001 | |
| Year-1 | 29.00(1.15) | 26.68(2.14) | 26.55(2.39) | *P*(MCI_A+V−_, MCI_A+V+_) = 0.538 | |
| Year-2 | 28.97(1.64) | 25.44(2.74) | 27.00(2.74) |  | |
| Year-4 | 29.07(1.64) | 24.73(2.63) | 24.55(4.39) |  | |
| **AVLT trials 1–5** |  |  |  | *P*(CU_A−V−,_  MCI_A+V−_) = 0.006 | |
| Baseline | 48.40(11.11) | 30.48(7.61) | 31.13(8.87) | *P*(CU_A−V−_, MCI_A+V+_) < 0.001 | |
| Year-1 | 48.33(11.61) | 29.32(8.87) | 30.86(7.16) | *P*(MCI_A+V−_, MCI_A+V+_) = 0.123 | |
| Year-2 | 49.56(10.20) | 29.72(9.85) | 26.67(8.94) |  | |
| Year-4 | 51.26(12.45) | 26.21(9.83) | 23.91(10.04) |  | |
| **AVLT delayed recall** |  |  |  | *P*(CU_A−V−,_  MCI_A+V−_) < 0.0001 | |
| Baseline | 8.15(4.28) | 2.31(2.70) | 3.61(3.10) | *P*(CU_A−V−_, MCI_A+V+_) < 0.0001 | |
| Year-1 | 8.57(4.64) | 2.00(2.55) | 2.73(3.33) | *P*(MCI_A+V−_, MCI_A+V+_) = 0.883 | |
| Year-2 | 8.81(4.01) | 2.21(2.92) | 1.44(1.92) |  | |
| Year-4 | 10.30(4.09) | 1.21(2.26) | 1.36(1.96) |  | |
| **Log-transformed TMT-A** |  |  |  | *P*(CU_A−V−,_  MCI_A+V−_) = 0.079 | |
| Baseline | 1.49(0.17) | 1.54(0.12) | 1.69(0.17) | *P*(CU_A−V−_, MCI_A+V+_) = 0.005 | |
| Year-1 | 1.44(0.14) | 1.57(0.15) | 1.67(0.14) | *P*(MCI_A+V−_, MCI_A+V+_) = 0.113 | |
| Year-2 | 1.46(0.14) | 1.53(0.16) | 1.69(0.20) |  | |
| Year-4 | 1.46(0.14) | 1.52(0.15) | 1.75(0.19) |  | |
| **Log-transformed TMT-B** |  |  |  | *P*(CU_A−V−,_  MCI_A+V−_) = 0.033 | |
| Baseline | 1.86(0.18) | 1.95(0.21) | 2.12(0.21) | *P*(CU_A−V−_, MCI_A+V+_) < 0.001 | |
| Year-1 | 1.85(0.15) | 1.95(0.23) | 2.16(0.20) | *P*(MCI_A+V−_, MCI_A+V+_) = 0.046 | |
| Year-2 | 1.83(0.18) | 1.97(0.24) | 2.22(0.20) |  | |
| Year-4 | 1.82(0.14) | 1.95(0.17) | 2.16(0.22) |  | |
| **SVF (animal)** |  |  |  | *P*(CU_A−V−,_  MCI_A+V−_) < 0.001 | |
| Baseline | 20.60(5.42) | 16.24(4.32) | 16.04(5.81) | *P*(CU_A−V−_, MCI_A+V+_) = 0.019 | |
| Year-1 | 21.30(4.69) | 15.64(4.81) | 15.91(5.09) | *P*(MCI_A+V−_, MCI_A+V+_) = 0.393 | |
| Year-2 | 21.59(4.98) | 16.21(5.19) | 15.56(5.16) |  | |
| Year-4 | 21.04(4.98) | 12.29(5.44) | 13.91(5.07) |  | |
| Note: Values are expressed as mean (standard deviation).  Abbreviations: AVLT = Auditory Verbal Learning Test; CU = cognitively unimpaired; MCI = mild cognitive impairment; MMSE = Mini-Mental State Examination; SVF = Semantic Verbal Fluency; TMT = Trail Making Test.  ^*^Year 1 = 62 participants (25 CU_A−V−_, 20 MCI_A+V−_, and 17 MCI_A+V+_), Year 2 = 65 participants (30 CU_A−V−_, 18 MCI_A+V−_, and 17 MCI_A+V+_), Year 4 = 27 participants (10 CU_A−V−_, 11 MCI_A+V−_, and 6 MCI_A+V+_). | | | | |  |

**Supplementary Material 3**


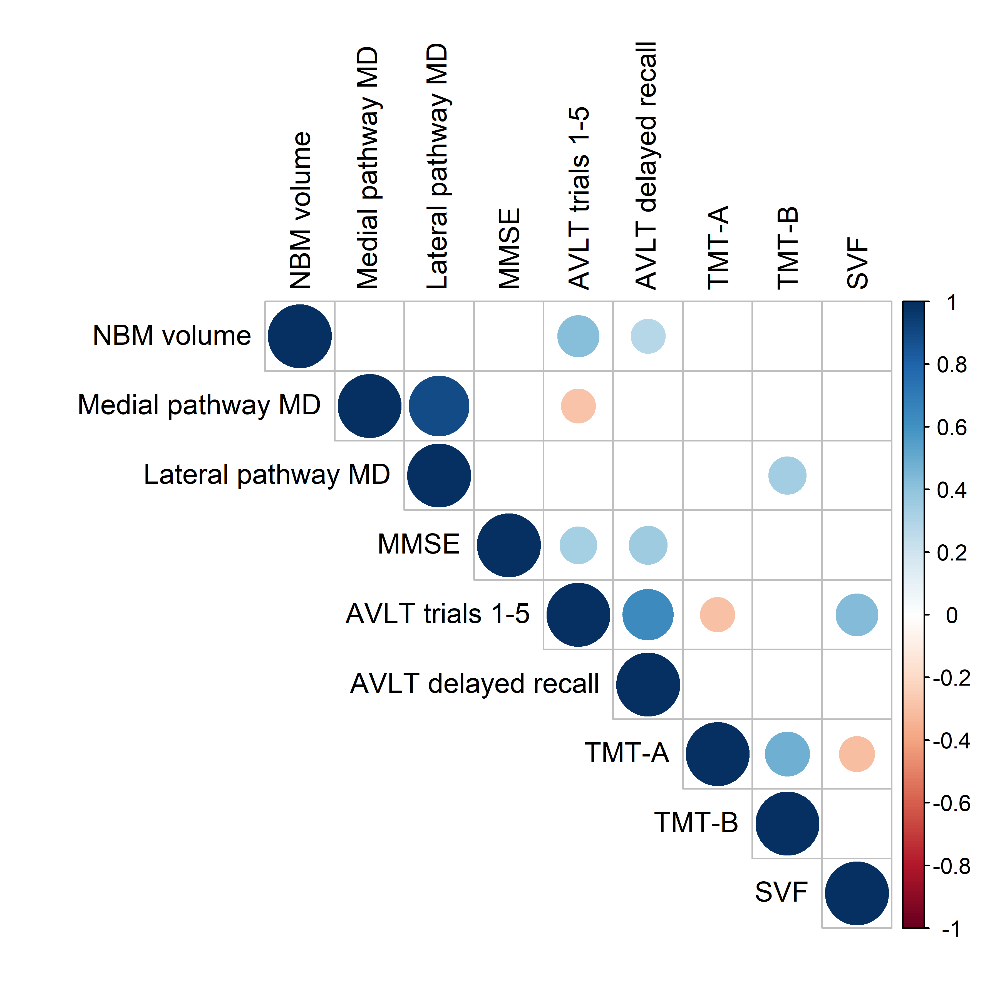


**Figure S5. Correlations between cholinergic system and cognitive performance.** Correlation coefficient is represented by color. Abbreviations: AVLT = Auditory Verbal Learning Test; MD = mean diffusivity; MMSE = Mini-Mental State Examination; NBM = nucleus basalis of Meynert; SVF = Semantic Verbal Fluency; TMT-A = Trail Making Test, Part A; TMT-B = Trail Making Test, Part B.

| **Supplementary Material 4.**  **Table S4. Longitudinal changes of cholinergic markers among groups.** | | | | |
| --- | --- | --- | --- | --- |
| **Variables**^*^ | **CU_A−V−_** | **MCI_A+V−_** | **MCI_A+V+_** | ***P*-value** |
| **Normalized NBM volume** |  |  |  | *P*(CU_A−V−,_  MCI_A+V−_) = 0.141 |
| Baseline | 0.18(0.02) | 0.16(0.03) | 0.15(0.02) | *P*(CU_A−V−_, MCI_A+V+_) = 0.141 |
| Year-1 | 0.16(0.03) | 0.13(0.03) | 0.13(0.02) | *P*(MCI_A+V−_, MCI_A+V+_) = 0.921 |
| Year-2 | 0.15(0.02) | 0.13(0.03) | 0.13(0.02) |  |
| Year-4 | 0.15(0.03) | 0.13(0.02) | 0.11(0.02) |  |
| **Medial pathway MD** |  |  |  | *P*(CU_A−V−,_  MCI_A+V−_) = 0.024 |
| Baseline | 0.80(0.04) | 0.81(0.04) | 0.85(0.03) | *P*(CU_A−V−_, MCI_A+V+_) = 0.003 |
| Year-1 | 0.81(0.03) | 0.82(0.03) | 0.87(0.03) | *P*(MCI_A+V−_, MCI_A+V+_) = 0.151 |
| Year-2 | 0.81(0.06) | 0.83(0.04) | 0.88(0.02) |  |
| Year-4 | 0.82(0.04) | 0.84(0.03) | 0.90(0.03) |  |
| **Lateral pathway MD** |  |  |  | *P*(CU_A−V−,_  MCI_A+V−_) = 0.085 |
| Baseline | 0.78(0.03) | 0.78(0.03) | 0.82(0.03) | *P*(CU_A−V−_, MCI_A+V+_) = 0.038 |
| Year-1 | 0.78(0.03) | 0.78(0.03) | 0.83(0.03) | *P*(MCI_A+V−_, MCI_A+V+_) = 0.316 |
| Year-2 | 0.78(0.05) | 0.79(0.04) | 0.84(0.03) |  |
| Year-4 | 0.79(0.04) | 0.80(0.04) | 0.86(0.05) |  |
| Note: Values are expressed as mean (standard deviation). Abbreviations: CU = cognitively unimpaired; MCI = mild cognitive impairment; MD = mean diffusivity; NBM = nucleus basalis of Meynert.  ^*^Year 1 = 62 participants (25 CU_A−V−_, 20 MCI_A+V−_, and 17 MCI_A+V+_), Year 2 = 65 participants (30 CU_A−V−_, 18 MCI_A+V−_, and 17 MCI_A+V+_), Year 4 = 27 participants (10 CU_A−V−_, 11 MCI_A+V−_, and 6 MCI_A+V+_). | | | | |

**Supplementary Material 5**

**
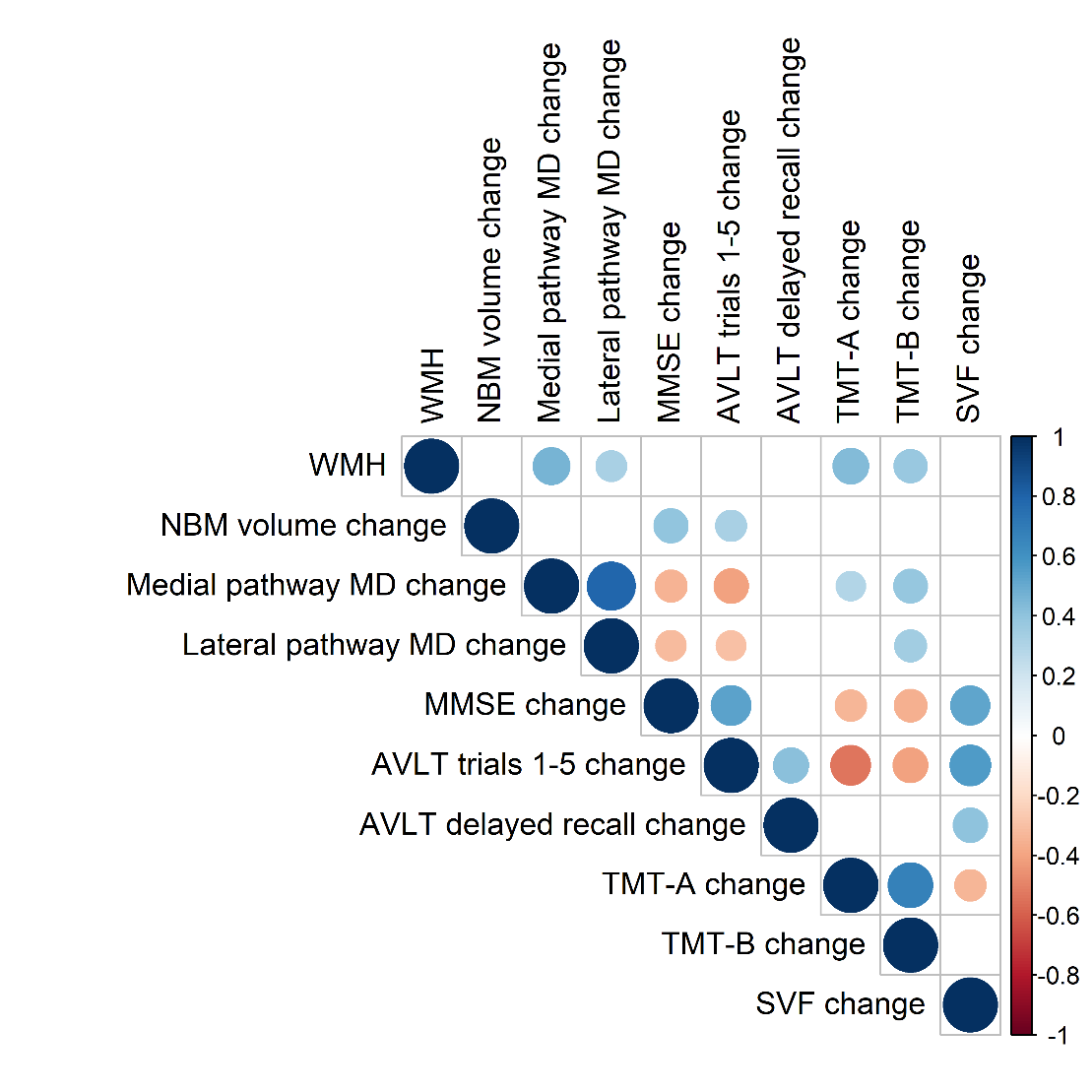
**

**Figure S6. Correlations between WMH burden, cholinergic system changes, and cognitive performance changes.** Correlation coefficient is represented by color. Abbreviations: AVLT = Auditory Verbal Learning Test; MD = mean diffusivity; MMSE = Mini-Mental State Examination; NBM = nucleus basalis of Meynert; SVF = Semantic Verbal Fluency; TMT-A = Trail Making Test, Part A; TMT-B = Trail Making Test, Part B; WMH = white matter hyperintensities.
